# A simplified Monte Carlo algorithm considering large‐angle scattering for fast and accurate calculation of proton dose

**DOI:** 10.1002/acm2.12221

**Published:** 2017-11-27

**Authors:** Taisuke Takayanagi, Shusuke Hirayama, Shinichiro Fujitaka, Rintaro Fujimoto

**Affiliations:** ^1^ Hitachi, Ltd., Research & Development Group Center for Technology Innovation – Energy Hitachi Japan

**Keywords:** dose calculation, low dose halo, proton beam therapy, simplified Monte Carlo

## Abstract

**Purpose:**

The purpose of this study is to improve dose calculation accuracy of the simplified Monte Carlo (SMC) algorithm in the low‐dose region. Because conventional SMC algorithms calculate particle scattering in consideration of multiple Coulomb scattering (MCS) only, they approximate lateral dose profiles by a single Gaussian function. However, it is well known that the low‐dose region spreads away from the beam axis, and it has been pointed out that modeling of the low‐dose region is important to calculated dose accurately.

**Methods:**

A SMC algorithm, which is named modified SMC and considers not only MCS but also large angle scattering resembling hadron elastic scattering, was developed. In the modified SMC algorithm, the particle fluence varies in the longitudinal direction because the large‐angle scattering decreases residual range of particles in accordance with their scattering angle and tracking of the particles with large scattering angle is terminated at a short distance downstream from the scattering. Therefore, modified integrated depth dose (m‐IDD) tables, which are converted from measured IDD in consideration of the fluence loss, are used to calculate dose.

**Results:**

In the case of a 1‐liter cubic target, the calculation accuracy was improved in comparison with that of a conventional algorithm, and the modified algorithm results agreed well with Geant4‐based simulation results; namely, 98.8% of the points satisfied the 2% dose/2 mm distance‐to‐agreement (DTA) criterion. The calculation time of the modified SMC algorithm was 1972 s in the case of 4.4 × 10^8^ particles and 16‐threading operation of an Intel Xeon E5‐2643 (3.3‐GHz clock).

**Conclusions:**

An SMC algorithm that can reproduce a laterally widespread low‐dose region was developed. According to the comparison with a Geant4‐based simulation, it was concluded that the modified SMC algorithm is useful for calculating dose of proton radiotherapy.

## 1 INTRODUCTION

Proton beams have a Bragg peak in their longitudinal profile and allow good dose localization; consequently, to take advantage of this feature, many proton‐therapy facilities have been planned and built. To distribute a finer dose effectively, some facilities use a technique called “pencil‐beam scanning” (PBS).^1^, ^2^ The dynamics of producing a dose field by PBS are different from those of “passive scattering” such as double scattering using rotating range modulators. The dose is deposited, inside the target in all three dimensions, as “dose spots” by scanning a focused pencil beam. By superposing a large number of individual dose spots, it is possible to optimize the dose given to the target.

The energy, irradiation position (spot position), and number of monitor units (MUs) of each pencil beam are optimized, and a three‐dimensional dose distribution is calculated by treatment‐planning software, which generally uses a pencil‐beam algorithm (PBA) for calculating dose.^3^, ^4^ As an example of a PBA, the fluence‐dose model^3^ calculates dose distribution by convolving in‐air fluence with the dose kernel. While a PBA can calculate dose quickly and its accuracy is clinically sufficient in most cases, it does not model edge scattering correctly and might produce errors in the boundary region of heterogeneous media having an edge parallel to the beam's central axis. Such heterogeneous effects have been analyzed using Monte Carlo (MC) techniques^3^, ^4^ because MC is superior to PBA in the aspect of particle scattering.

MC techniques calculate individual particle tracks in order to obtain a dose at a certain point. They can therefore accurately simulate the edge‐scattering effect on proton treatment planning. MC techniques have previously been applied for proton treatment planning by using well‐established software packages such as Geant4, FLUKA, and MCNPX.^5^, ^6^, ^7^, ^8^, ^9^ However, they require a long calculation time and are difficult to use for daily routine treatment.

Possible methods proposed to speed up MC techniques include parallel computation^10^, ^11^, ^12^, ^13^, ^14^ and model simplification.^15^, ^16^ For the latter, Fippel and Soukup^15^ used a simplified model of proton‐material interactions. For instance, with this model, stopping power and radiation length of materials are simply determined on the basis of comparisons with those properties of water. Moreover, nuclear interactions are simulated in consideration of the cross‐sections of hydrogen and oxygen only. While the results of dose calculations based on their simplified model agree well with those obtained with Geatn4, the calculation speed is 23 times faster than that of Geant4. Li et al.^16^ developed a track‐repeating algorithm for proton therapy that maintains the accuracy of the MC technique, while significantly decreasing computation times. The calculation speed for a dose in heterogeneous media is about ten times faster than that of Geant4. The algorithm utilizes a pregenerated database of histories of particles produced when water is irradiated with protons, and dose distributions in heterogeneous anatomies are calculated by retracing the proton tracks.

Thanks to its two merits (explained in the following), the simplified Monte Carlo algorithm^17^, ^18^, ^19^ (hereafter, SMC) is focused on in the present study. The first merit is, of course, its simplicity. Since the SMC utilizes preset tables of integrated depth dose (IDD), as is the case with the PBA, dose at a certain point is obtained quickly without the need of a complex calculation. The second merit is that the commissioning procedure is the same as that used by the PBA. IDD tables and the initial‐phase space parameters used for the PBA might be applicable to the SMC without major adjustment. As is the case with other MC techniques, the SMC calculates individual particle tracks. It can therefore accurately simulate the edge‐scattering effect on proton treatment planning. It has been reported that the SMC can calculate proton‐dose distribution in heterogeneous media accurately in a calculation time of about 20 min.^18^

Conventional SMC algorithms calculate particle scattering in consideration of multiple Coulomb scattering (MCS) only.^17^, ^18^, ^19^ The calculated lateral‐dose profiles are therefore approximated by a single Gaussian function. However, it is well‐known that the low‐dose region spreads away from the beam axis, and it has been previously pointed out that the modeling of the low‐dose region is an important factor in accurately calculating dose deposited by PBS.^20^, ^21^, ^22^, ^23^, ^24^, ^25^, ^26^, ^27^ It is reported that dose calculation algorithms with insufficient model of the low‐dose region require much effort for the beam modeling, which is a procedure for adjusting phase‐space parameters in order to keep the dose calculation accuracy.

In the present study, an SMC algorithm, which is named modified SMC and considers not only MCS but also large angle scattering resembling hadron elastic scattering and can reproduce a laterally widespread low‐dose region was developed. Essentially, a lot of physical phenomena contribute to reproduction of the low‐dose region. Protons scattered at a large angle contribute only part of the dose in the low‐dose region. In the modified SMC algorithm, it is considered that these scattered primary protons behave as virtual particles which have contribution of secondary particles also.

In the rest of this paper, the basis of the conventional SMC algorithm is first introduced. Second, the features of the modified SMC algorithm are described. Finally, to evaluate the usefulness of the modified SMC algorithm, the calculated dose distributions are compared with those obtained by using a Geant4‐based simulation. In present study, the modified SMC algorithm was developed from scratch by using C++.

## 2 MATERIALS AND METHODS

### 2.A Conventional SMC algorithm

**Figure 1:**
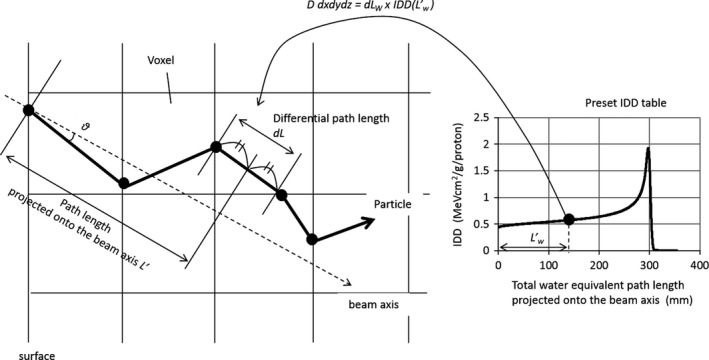
Schematic drawing of particle tracking by conventional SMC algorithms.

The modified SMC algorithm basically follows conventional SMC algorithms as following. A schematic drawing of particle tracking by conventional SMC algorithms is shown in Fig. 1. In media divided into voxels, particles travel in a straight line on the basis of the given momentum direction. At the border of voxels, particle information (residual range *R* and momentum direction) is updated on the basis of the differential water‐equivalent path length d*L*_*W*_, which is derived on the basis of water‐equivalent ratio (WER) of the voxel and differential path length d*L* in Euclidean space as

(1) $$dL_{w}=WER\times dL.$$

As is the case with the PBA, the computed tomography (CT) value of each voxel is converted to WER by using a calibrated conversion table.

Residual range *R* of a particle passing through *n* voxels is given as

(2) $$R=R_{0}-L_{w}=R_{0}-\sum_{j}^{n}dL_{W}\left(j\right)=R_{0}-\sum_{j}^{n}WER\left(j\right)\times dL\left(j\right)$$

where *R*_*0*_ is initial residual range of a particle and *L*_*W*_ is total water‐equivalent path length. The particle energy is also derived from *R*. When *R* falls below a cut‐off value, the particle tracking is terminated, and the tracking for the next particle starts. Momentum direction is changed by MCS.

SMC algorithms can rapidly calculate dose *D*_*i*_ given from *i*th particle*,* by using preset IDD tables. *D*_*i*_ in a voxel at a given position $\vec{x}$ is given as:

(3) $$D_{i}\left(\vec{x}\right)dxdydz=dL_{w}\times IDD\left(L_{w}^{\prime }\right)$$

where *dx, dy,* and *dz* are width of the voxel. *L’*_*W*_ is total water‐equivalent path length projected onto beam axis, which is a straight line passing through the beam center of each dose spot and is determined by the deflection angle of scanning magnets. Therefore, total dose *D* in the voxel is given as:

(4) $$D\left(\vec{x}\right)dxdydz=IDD\left(L{{}^{\prime }}_{w}\right)\sum_{i=1}^{N(\vec{x})}dL_{\mathrm{wi}}\left(\vec{x}\right)$$

where *N(* $\vec{x}$ *)* is the number of protons passing through a given position $\vec{x}$, and *dL*_wi_ is the differential water‐equivalent path length of the *i*th particle. When *z* direction is equal to the beam axis, IDD at a given depth *z* is defined as

(5) $$IDD\left(z\right)\equiv \frac{1}{N_{0}}\int_{-\infty }^{\infty }\int_{-\infty }^{\infty }D_{\mathrm{single}}\left(\vec{x}\right)dxdy$$

where *N*_*o*_ is number of incident protons. *D*_*single*_ is dose distribution in water, produced by a mono‐energetic single pencil beam. IDD of each energy is measured by using water tanks and large‐area ion chambers. In general, because the radius of ion chambers is limited, measured IDD requires correction by other MC, and the shape is adjusted.

An independent variable in the IDD tables is total water‐equivalent path length projected onto beam axis *L’*_*W*_. Here, *L’*_*W*_ of a particle passing through *n* voxels is given as

(6) $$L_{w}^{\prime }=\frac{dL_{W}\left(n\right)\cos\theta_{n}}{2}+\sum_{j}^{n-1}dL_{W}\left(j\right)\cos\theta_{j}$$

where *θ*_*j*_ is the angle between the momentum direction in voxel *j* and the beam axis. In the SMC algorithms, from the viewpoint of law of energy conservation, particles passing through an area at a certain water‐equivalent depth must give the same dose into media. Moreover, the particle fluence must be invariant in each water‐equivalent depth. Namely, in conventional SMC algorithms, fluence loss of primary particles and generation of secondary particles are not considered.

### 2.B Modified SMC algorithm

**Figure 2:**
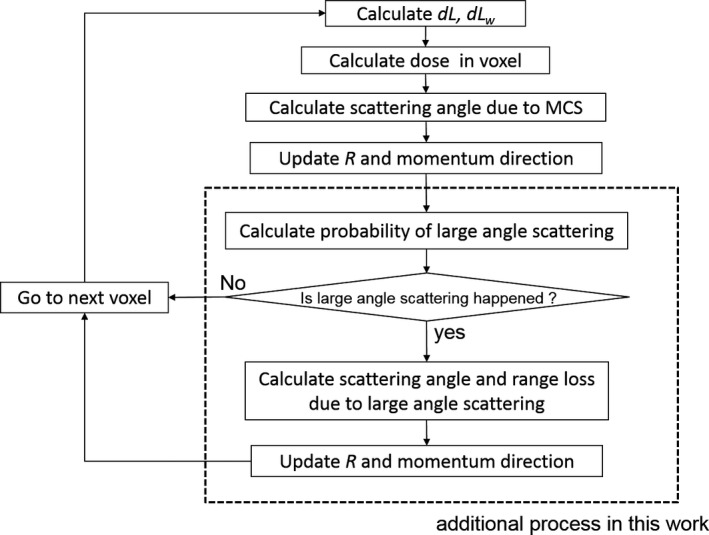
Flowchart of the modified SMC algorithm.

Figure 2 shows a flowchart of the modified SMC algorithm. The modified SMC algorithm basically follows conventional SMC algorithms. Key changes in the modified SMC algorithms are described here.

#### 2.B.1 Particle scattering

In order to reproduce laterally widespread low‐dose region, the modified SMC algorithm considers not only MCS but also large‐angle scattering. As described above, momentum variation due to MCS are calculated at the border of voxels. In the modified SMC algorithm, the standard deviation *σ* of the scattering angle for MCS is calculated by using the empirical formula proposed by Lynch and Dahl.^28^

**Figure 3:**
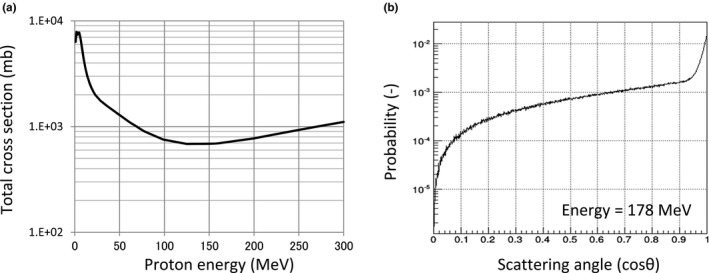
(a) The total cross‐section of large‐angle scattering, and (b) scattering angle of 178 MeV protons in the modified SMC algorithm. The total cross‐section was obtained by summing the total cross‐section of proton‐hydrogen scattering and that of proton‐oxygen scattering, provided by G4HadronElastic model. Before the summation, the cross‐sections of these scattering were adjusted by multiplying a factor depending on proton energy.

After calculating MCS, it is decided whether large‐angle scattering occurs or not. The probability *P* is given as

(7) $$P=M^{-1}N_{a}\rho_{w}\times dL_{w}\times \sigma_{\mathrm{LAS}}$$

where *M* is mole number of water, *N*_*a*_ is Avogadro number, *ρ*_w_ is density of water, and *σ*_*LAS*_ is the total cross‐section of large‐angle scattering. Figure 3(a) shows *σ*_*LAS*_ used in the modified SMC algorithm. Moreover, as representative, angular distributions of 178.2 MeV protons are shown in Fig. 3(b). The total cross‐section and the angular distribution are calculated on the basis of G4HadronElastic model, which is a hadron‐nucleus elastic scattering model provided by the Geant4^9^ version 9.3. In the case of calculating proton‐nucleolus interaction, this model is a parameterization of experimental data.

In the modified SMC, scattering in water was only considered. The total cross‐section shown in Fig. 3(a) was obtained by summing the total cross‐section of proton‐hydrogen scattering *σ*_*p‐H*_ and that of proton‐oxygen scattering *σ*_*p‐O*_, provided by G4HadronElastic model;

(8) $$\sigma_{\mathrm{LAS}}=2F_{p-H}\times \sigma_{p-H}+F_{p-O}\times \sigma_{p-O}.$$

Before the summation, the cross‐sections of these scattering are adjusted by multiplying factors *F*_*p‐H*_ and *F*_*p‐O*_ depending on proton energy. Strictly speaking, the low‐dose region is due to not only elastic scattering but also a lot of physical phenomena (such as inelastic scattering and secondary particles). Therefore, in the modified SMC, the total cross‐sections provided by the G4 Hadron Elastic model should be adjusted in order to simulate the low‐dose region by contributions from the elastic scattering only. This factor was decided as the modified SMC can reproduce dose distributions of mono‐energetic single pencil beam calculated by a full Monte Carlo (FMC) simulation. The correction factor *F*_*p‐H*_ and *F*_*p‐O*_ are shown in Table 1. The factors for energies that are not given in the table are linearly interpolated. In high energy, the correction factor *F*_*p‐H*_ reaches seventeen. It indicates that contributions from inelastic interaction and secondary particles are dominate in the low‐dose region in comparison with primary particle scattered with large angle.

**Table 1:** Correction factors for the total cross‐section derived by G4HadoronElasitc at three energies

	10 MeV	100 MeV	250 MeV
*F*_*p‐O*_	1.0	1.0	1.0
*F*_*p‐H*_	3.0	8.0	17

In the present study, to maintain calculation speed and simplicity of the modified SMC algorithm, the low‐dose region was reproduced by implementing large‐angle scattering of primary protons only. As mentioned above, essentially, a lot of physical phenomena (such as large‐angle scattering of primary and secondary protons, secondary neutrons, delta rays, and gamma rays) contribute to reproduction of the low‐dose region. Moreover, large‐angle scattering of protons is due to a combination of elastic and inelastic nuclear reactions. Namely, primary protons scattered at a large angle contribute only part of the dose in the low‐dose region. In the modified SMC algorithm, it is considered that these scattered primary protons behave as virtual particles which have contribution of secondary particles also.

In the modified SMC algorithm, the large‐angle scattering is regarded as elastic interaction; that is, residual range of particles decreases not only based on eq. (2) but also in accordance with their scattering angle. Since tracking of the particles with large scattering angle is terminated at a short distance downstream from the scattering, the particles fluence decreases in the longitudinal direction.

#### 2.B.2 Preset table for dose calculation

The modified SMC algorithm cannot use IDD tables directly for the dose calculation because the particles fluence is not invariant in the longitudinal direction. Adequate preset tables, called modified IDD (m‐IDD), is required and converted from IDD. The conversion factor *C(z)* is derived as following. In the dose calculation of single pencil beam, the modified SMC algorithm must satisfy the following equation:

(9) $$D_{\mathrm{single}}\left(\vec{x}\right)dxdydz=m\_IDD\left(L{{}^{\prime }}_{w}\right)\sum_{i=1}^{N(\vec{x})}dL_{\mathrm{wi}}\left(\vec{x}\right).$$

By integrating with respect to *x* and *y*, the eq. (9) becomes

(10) $$\frac{1}{N_{0}}\int \;\;\;\int D_{\mathrm{single}}\left(\vec{x}\right)dxdy=\frac{m\_IDD(L{{}^{\prime }}_{w})}{dzN_{0}}\sum_{i=1}^{N(z)}dL_{\mathrm{wi}}\left(z\right)=IDD\left(z\right).$$

Therefore, the following relations can be derived:

(11) $$m\_IDD\left(z\right)=C\left(z\right)IDD\left(z\right)$$

(12) $$C^{-1}\left(z\right)=\frac{\left[\sum_{i=1}^{N(z)}dL_{\mathrm{wi}}\left(z\right)\right]}{N_{0}dz}≅\frac{N(z)}{N_{0}}$$

Equation (12) indicates that the inverse of *C(z)* is almost equivalent to the particle survival fraction at depth *z*. The quantity in square brackets in eq. (12) can be computed by the modified SMC algorithm if all preset dose values are set to one.

### 2.C Evaluation of the modified SMC algorithm

#### 2.C.1 Calculation speed

Calculation time for 500,000 particles is evaluated. Infinitesimal proton beams, with emittance of zero, are irradiated onto the surface of a water tank. The voxel size of the tank is 1 mm. Beam energies of 118, 178.2, and 218.9 MeV are evaluated. Single thread operation of an Intel Xeon E5‐2643 (3.3‐GHz clock) is used. The average computation time by repeating calculation five times are obtained and compared with that of the FMC. The simulation setup of the FMC, such as the voxel size, is same as that of the improved SMC. The physics model of the FMC is mentioned in the Section 2.C.2.

#### 2.C.2 Calculation accuracy

The accuracy of the dose calculation by the modified SMC was evaluated by comparing it with a FMC simulation. The FMC simulator was developed by using Geant4 library version 9.3. The Binary cascade model was used for the hadron interaction. The cut‐off value was 1 mm for secondary electron, positron, and gamma ray production.

**Figure 4:**
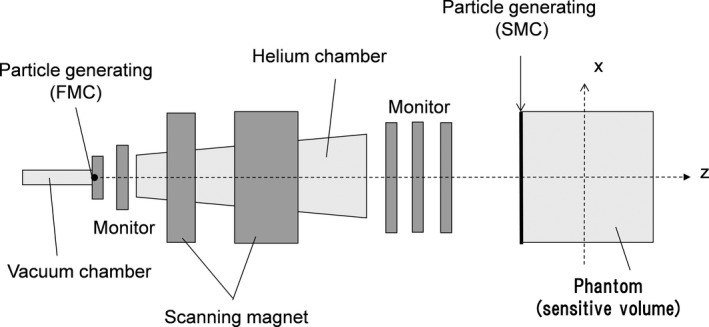
Schematic drawing of proton PBS nozzle.

A schematic drawing of the proton PBS nozzle considered in this study is shown in Fig. 4. In the FMC simulation, the particle tracking starts from the nozzle entrance (exit of the vacuum chamber). The particles pass through the nozzle components (vacuum chamber window, helium chamber, and monitors) and reach the phantom (a sensitive volume). Magnetic fields are generated by the two scanning magnets and scan particles on the basis of a given spot position.

On the other hand, in the modified SMC simulation, the particle tracking starts from the surface of the sensitive volume. The initial positions of particles are determined on the basis of a given spot position and phase‐space parameters of the pencil beam. Moreover, the initial particle directions are determined on the basis of the source‐axis distance (SAD) and the phase‐space parameters. SAD is about 2.5 m, which is equal to the distance from the center of the two scanning magnets to the isocenter.

The phase‐space parameters for the modified SMC algorithm are calculated by the FMC simulator. The lateral fluence distribution of the pencil beam is approximated by double Gaussian functions, and a sigma matrix and a weight are obtained for each Gaussian function. The procedure for obtaining the phase‐space parameters is explained in detail in the literature.^25^ IDD tables for the modified SMC algorithm are also calculated by the FMC simulator. The calculated IDD tables are then converted to m‐IDD tables by using the method described above.

Beam irradiation conditions for the evaluation are as follow:

1. Single‐pencil‐beam in homogeneous media

Three‐dimensional dose distributions by irradiation with a single‐pencil‐beam were evaluated. Generally, a beam which has a range of around 20 g/cm^2^ is used to evaluate a dose calculation algorithm.^29^ Therefore, in the present study, beam energies used were 118, 178.2, and 218.9 MeV with ranges of 10, 21, and 30 g/cm^2^ respectively.

- Scanned beam in homogeneous media

To evaluate dose calculation accuracy in a near‐clinical situation, dose distribution for scanned‐beam irradiation was evaluated. As is the case with the single‐pencil‐beam irradiation, beam energies were 118, 178.2, and 218.9 MeV. Field sizes of 5 × 5 cm^2^ and 10 × 10 cm^2^ were evaluated. Spot spacing was 5 mm.

Dose distribution in the case of a volumetric irradiation was also evaluated. The range and spread‐out Bragg peak (SOBP) were 30.6 g/cm^2^ and 10 cm, respectively, and field size was 10 × 10 cm^2^. That is, the target was a one‐liter cube. Spot spacing was 5 mm.

2D‐dose distributions is sampled from the Z‐X plane (including the isocenter), and the gamma indexes between the SMC algorithms and the FMC are evaluated. The criterion of the gamma evaluation is 2% dose/2 mm distance‐to‐agreement (DTA). For absolute dose comparison, the each dose calculated by the modified SMC and the FMC are normalized by the Bragg peak dose in the FMC. In the volumetric irradiation, the each dose are normalized by the isocenter dose (Z = 25.6 cm) in the FMC. For reducing statistical uncertainty, in the FMC, 6.1 × 10^7^ and 2.2 × 10^8^ particles are calculated in the field size of Field sizes of 5 × 5 cm^2^ and 10 × 10 cm^2^ respectively. In the modified SMC, 1.5 × 10^8^ and 5.3 × 10^8^ particles are calculated in the field size of Field sizes of 5 × 5 cm^2^ and 10 × 10 cm^2^ respectively.

- Scanned beam in heterogeneous media

**Figure 5:**
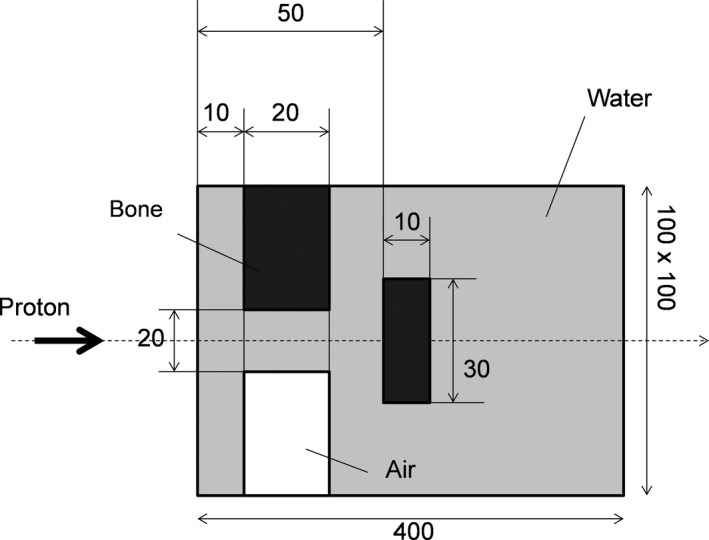
Setup for calculating dose in heterogeneous media (unit: mm).

The calculation setup is shown in Fig. 5. Two bone heterogeneities and one air heterogeneity (with water‐equivalent ratios of 2 and 0.001, respectively) were included in the homogeneous water. Mono‐energetic beams (with energies of 118, 178.2, and 218.9 MeV and ranges of 10, 21, and 30 g/cm^2^, respectively) were evaluated. Nominal field size was 5 × 5 cm^2^. Spot spacing was 5 mm.

2D‐dose distributions is sampled from the Z‐X plane (including the isocenter), and the gamma indexes between the SMC algorithms and the FMC are evaluated. The criterion of the gamma evaluation is 2% dose/2 mm DTA. The each dose calculated by the modified SMC and the FMC are normalized by the peak dose in the FMC. For reducing statistical uncertainty, 1.5 × 10^8^ and 6.1 × 10^7^ particles are calculated in the modified SMC and the FMC respectively.

## 3 RESULTS

### 3.A Converted m‐IDD tables

**Figure 6:**
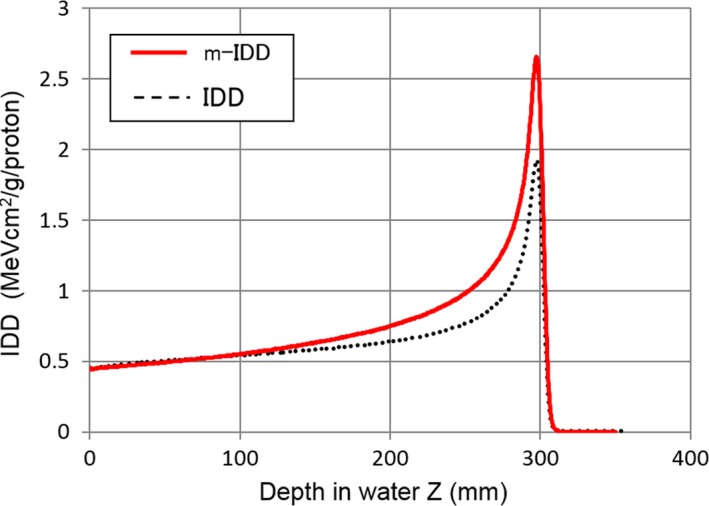
IDD and m‐IDD tables derived on the basis of eqs. (11) and (12).

m‐IDD tables converted from IDD on the basis of eqs. (11) and (12) is shown in Fig. 6. The results of 219.8 MeV are shown as representative. The peak of the m‐IDD became to be finer than that of the IDD. This trend is similar to that between linear energy transfer and IDD.

### 3.B Calculation speed

In comparison with the calculation speed of the FMC using Geant4, the calculation speed of the modified SMC was improved averagely by a factor 32.7. Calculation time of the modified SMC algorithm was equivalent to that of a conventional SMC algorithm. Although the calculation cost of the modified SMC algorithm is increased by inclusion of large‐angle scattering, decreasing total step length due to particle loss contributes to reducing calculation time.

### 3.C Calculation accuracy

#### 3.C.1 Single‐pencil‐beam irradiations in homogeneous media

**Figure 7:**
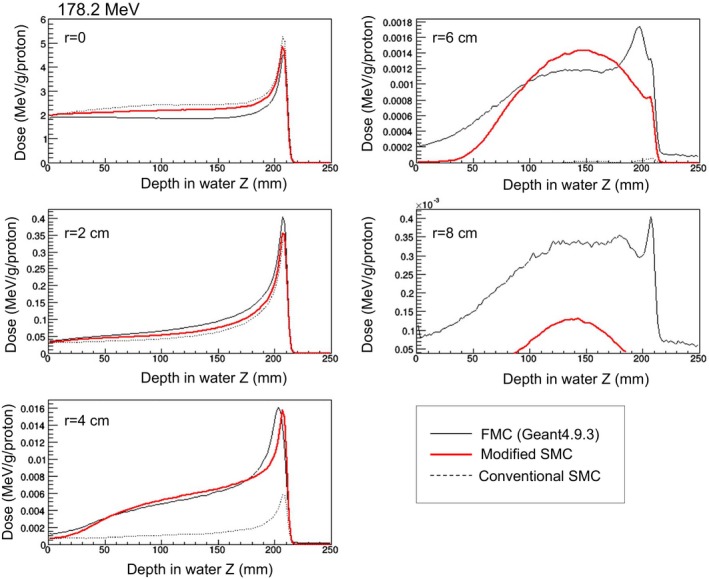
Calculated dose by single‐pencil‐beam irradiation (at 178.2 MeV).

Dose distributions in the case of single‐pencil‐beam irradiations are shown in Fig. 7. The results of 178.2 MeV are shown as representative. The horizontal axis is depth in water, the vertical axis is dose normalized by number of particles, and *r* is distance from the beam axis.

Especially for r = 4 cm, the calculation accuracy of the modified SMC algorithm was improved. Also, in the region of r < 2 cm, the calculation accuracy was improved slightly. For 218.9 MeV also, calculation accuracy of the modified SMC algorithm was improved. On the other hand, for 118 MeV, the modified SMC algorithm gave calculated doses that were almost the same as given by the conventional SMC algorithm.

#### 3.C.2 Scanned beam irradiations in homogeneous media

1. Mono‐energy irradiation

**Figure 8:**
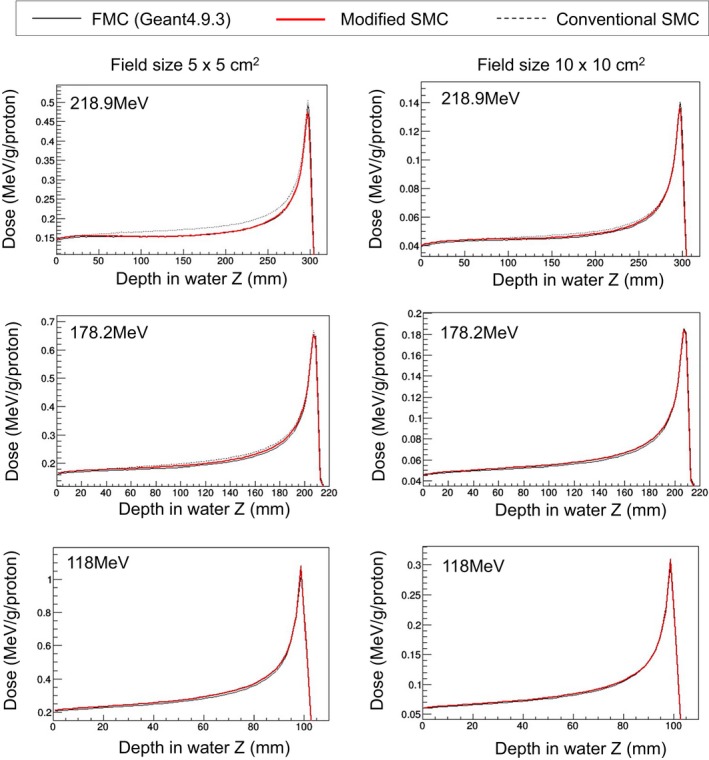
Calculated absolute depth dose by scanned beam irradiation.

Absolute depth dose (ADD) in the case of scanned‐pencil‐beam irradiation is plotted in Fig. 8. Each ADD was sampled from the center of the dose field. In each graph, the horizontal axis is depth in water, and the vertical axis is dose normalized by number of particles.

The modified SMC algorithm was effective in the case of higher beam energy and smaller field size. For proton energy of 218.9 MeV, the calculation accuracy of the modified SMC algorithm was improved in the case of both field sizes. For proton energy of 178.2 MeV also, for the field size of 5 × 5 cm^2^, improvement was observed. However, for the field size of 10 × 10 cm^2^, the conventional SMC algorithm already had agreement with the FMC, and the modified SMC algorithm showed slight improvement. Moreover, for proton energy of 118 MeV, there was a slight difference among calculation accuracies of these three algorithms for both field sizes.

**Figure 9:**
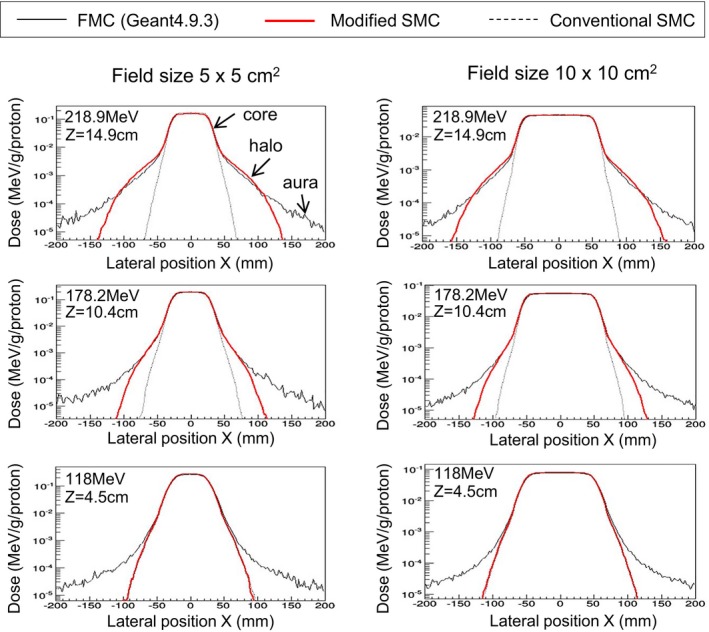
Calculated OCR in middle range by scanned beam irradiation. Core is dose due to particles which have only undergone MCS, namely dose calculated by the conventional SMC algorithm.

Off‐center ratio (OCR) for the scanned‐pencil‐beam irradiation is plotted in Fig. 9, in which the horizontal axis is the lateral position, and the vertical axis is dose normalized by the number of particles. These graphs plot OCR at the middle range.

For proton energy of 218.9 MeV, the calculation accuracy of the modified SMC algorithm was improved; the dose bump, namely, the halo was reproduced accurately. On the other hand, the halo decreased and the difference with the conventional algorithm became small for the low energy beam. Although this seems to indicate an insufficiency of the modified SMC algorithm, it is merely that the dose weight of halo is essentially small in the low energy beam. This point is also supported by the results that the differences between the three algorithms (conventional SMC, modified SMC, and FMC) became small for the proton energy of 118 MeV (see Fig. 8).

The dose in the regions far away from the center, namely, the aura was not reproduced by the modified SMC algorithm. This indicates that there is room of improvement for the modified SMC algorithm. However, according to Fig. 7, it is consider that the influence of the aura would be slight, and the simulation of the aura is unnecessary.

**Figure 10:**
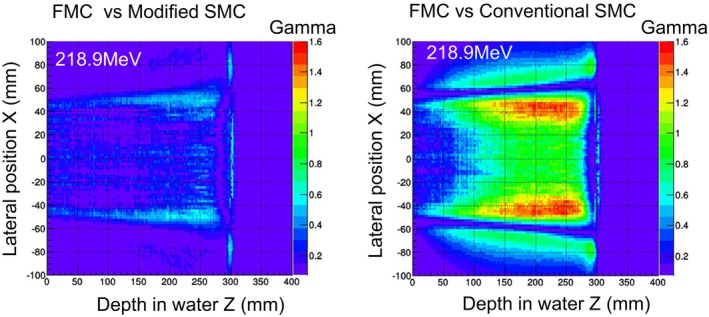
Results of gamma evaluation between Geant4‐based FMC and SMC algorithms.

The gamma indexes, given by comparing the SMC algorithms to the FMC are shown in Fig. 10. The results of the 218.9 MeV beam in the field size of 10 × 10 cm^2^ are shown as representative. The horizontal axis is depth in water, and the vertical axis is the lateral position. In comparison with the conventional SMC algorithm, the modified SMC algorithm gave an improved dose profile especially at the edge of the dose field and in the proximal region.

The pass rates, for which the low dose threshold is 20%, are listed in Table 2. The dose distributions calculated by the modified SMC algorithm agreed well with those given by the FMC simulation; namely, the pass rate was almost 100%. The pass rate of the conventional SMC algorithm decreased in the case of small dose field and high‐energy proton beam. This trend was consistent with the results in Fig. 8.

**Table 2:** Pass rate (%) of gamma evaluation between Geant4‐based FMC and SMC algorithms

		118 MeV	178.2 MeV	218.9 MeV
Conventional SMC	5 × 5 cm^2^	99.2	69.0	39.8
	10 × 10 cm^2^	97.9	97.2	76.4
Modified SMC	5 × 5 cm^2^	99.2	100	100
	10 × 10 cm^2^	97.7	100	100

- Volumetric irradiation

**Figure 11:**
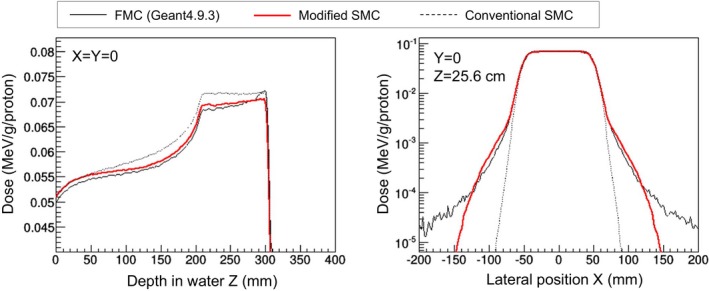
Calculated ADD and OCR in the case of volumetric irradiation for a 1‐liter cubic target.

Calculated ADD and OCR are shown in Fig. 11 for volumetric irradiation for a one‐liter cubic target. The left graph plots ADD sampled from the center of the field, and the right one plots OCR sampled from the isocenter. The dose was normalized by the number of particles. Calculation time of the modified SMC algorithm was 1972 s in the case of 4.4 × 10^8^ particles and 16‐threading operation of an Intel Xeon E5‐2643 (3.3‐GHz clock). The voxel size of the phantom was 2 mm. Calculation time of the conventional SMC algorithm was 1789 s for the same conditions.

ADD in the whole depth region calculated by the modified SMC algorithm was improved in comparison with that calculated by the conventional SMC algorithm (Fig. 11, left). It was especially improved in the proximal and SOBP regions. However, at the distal end, the calculated ADDs differed; that is, the small peak shown in the FMC‐simulation results was not reproduced in the modified SMC algorithm results. We considered that a slight overestimate of the halo dose caused this difference. As also shown in Fig. 9, the modified SMC algorithm reproduced the dose bump, namely, the halo (Fig. 11, right). Although the dose in the region far from the center of the dose field, namely, the aura, was not reproduced, this difference negligibly affected ADD.

**Figure 12:**
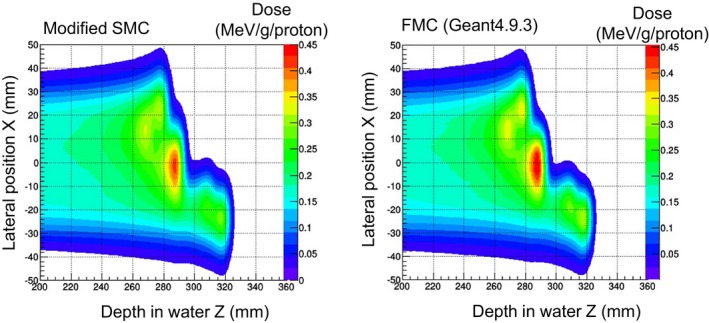
Results of gamma evaluation between Geant4‐based FMC and SMC algorithms.

The gamma indexes, given by comparing the SMC algorithms to the FMC are shown in Fig. 12. The horizontal axis is depth in water, and the vertical axis is lateral position. The dose distribution calculated by the modified SMC algorithm agreed well with that given by the FMC simulation. The pass rate at the threshold, which was the 20% dose, was 98.8%. For the conventional SMC algorithm, the pass rate decreased to 40.4%. As also shown in Fig. 11, the modified SMC algorithm gave an improved dose profile especially at the edge of the dose field and in the proximal region. On the other hand, at the distal end, the dose distribution calculated by the modified SMC algorithm still differed from that by the FMC simulation.

#### 3.C.3 Scanned beam irradiations in heterogeneous media

**Figure 13:**
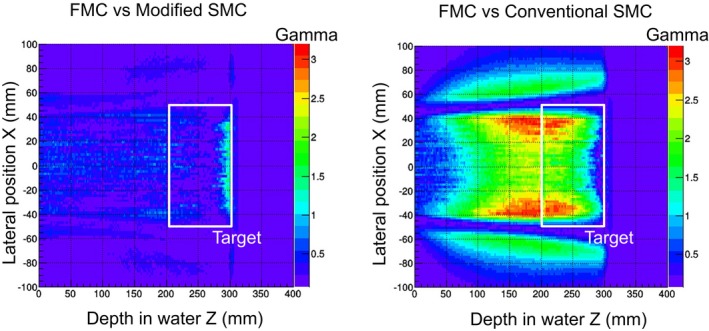
Calculated dose in heterogeneous media by the modified SMC algorithm and Geant4‐based simulation.

2D dose distributions calculated by the modified SMC algorithm are shown in Fig. 13. The horizontal axis is depth in water, and the vertical axis is lateral position. The results of the 218.9 MeV beam are shown as representative. The distributions were sampled from the Z‐X plane (including the isocenter). The dose was normalized by the number of particles. As previously reported,^16^, ^17^, ^18^ it is considered that the modified SMC algorithm can accurately calculate dose distributions in heterogeneous media.

For dose distributions in Fig. 13, quantitative evaluations using gamma index, given by comparing the SMC algorithms to the FMC, were also done. The pass rates at the threshold, which was the 20% dose, are listed in Table 3. The doses distributions calculated by the modified SMC algorithm agreed well with those calculated by the FMC simulation. As was the case with homogeneous water, the pass rate was almost 100%. On the other hand, the doses calculated by the conventional SMC algorithm differed from those calculated by the FMC simulation. Especially in the proximal region, the gamma index was increased in comparison with that of the modified SMC algorithm. These trends were similar to trends in the case of homogeneous water.

**Table 3:** Pass rate (%) of gamma evaluation between Geant4‐based FMC and SMC algorithms (in heterogeneous media)

	118 MeV	178.2 MeV	218.9 MeV
Conventional SMC	98.7	75.2	41.2
Modified SMC	99.5	99.9	99.9

## 4 DISCUSSION

In the case of lower energy beam or larger field, differences between the calculated doses given by the three algorithms (modified SMC, conventional SMC, and the FMC simulation) become small. This fact indicates that, in such a case, low‐dose region and particle scattering in media affects the dose distribution negligibly and the phase‐space parameters in air are dominant. Beam modeling, which is a procedure for adjusting phase‐space parameters, would become more important in regard to calculating dose accurately. The modified SMC algorithm is effective for higher beam energy and smaller field size.

According to Figs. 9 and 11, differences from FMC still remain in the regions far away from the center, namely, the aura, and these differences affect dose distributions slightly in the center of the field. This indicates that there is room for improvement in the modified SMC algorithm. In the case of single‐pencil‐beam irradiation, moreover, the difference between calculated dose distributions is also shown in the r = 0 region, namely, the core. That difference indicates that the MCS model also has room for improvement. In the future, we will investigate the impacts of these differences in clinical situations. However, we consider that clinically acceptable calculation accuracy would be achieved by just adjustment of phase‐space parameters, and big modifications would not be required for the modified SMC algorithm. In clinically utilized treatment‐planning software using PBA, adjustment of the phase space parameters is a popular technique for achieving adequate calculation accuracy without modification of the dose kernel. Since it is difficult to construct dose kernel perfectly simulating dose distribution of infinitesimal beam, the treatment‐planning software finally ensures the calculation accuracy by adjustment of the phase space parameters.^25^ Here, modification of dose kernel in PBA is equivalent to modification of the dose calculation algorithm in the modified SMC. When adjustment of phase‐space parameters does not work adequately to ensure the calculation accuracy, we should investigate whether the dose distribution difference can be suppressed by adjusting the probability of large‐angle scattering and the angular distribution of protons. Alternatively, to suppress the difference, a stricter physics model might be considered.

The advantage of the modified SMC algorithm is the low dependency of the calculation environment because the speed‐up is achieved by the algorithm design only. Commercial treatment‐planning software systems would be expected to be installed in many types of computers and to run with the maximum performance without special tuning. Moreover, the modified SMC algorithm has room for GPU acceleration. (The environment independency might be lost.) According to a previous study,^30^ the modified SMC algorithm can be expected to be further improved by a factor of 20. We believe that further speed‐up is valuable for dose calculations requiring more speed, such as adaptive radiotherapy, and creating a dose matrix for inverse planning considering robustness.

## 5 CONCLUSION

To achieve fast and accurate proton‐dose calculation for PBS, a SMC algorithm, which is named modified SMC and can reproduce a laterally widespread low‐dose region was developed. The calculation accuracy of the modified SMC algorithm was evaluated under the condition of proton irradiation by PBS. The results of the evaluation revealed that the modified SMC algorithm improved calculation accuracy in comparison with conventional SMC algorithms. The doses calculated by the modified SMC algorithm agreed well with those calculated by the FMC simulation based on Geant4. In the case of heterogeneous media also, dose distributions calculated by the modified SMC algorithm were consistent with those given by the FMC. It was therefore concluded that the modified SMC algorithm is useful for proton radiotherapy using PBS.

## CONFLICT OF INTEREST

Authors Taisuke Takayanagi, Shusuke Hirayama, Shinichiro Fujitaka, and Rintaro Fujimoto are employees of Hitachi, Ltd., Tokyo, Japan.
